# A New Law Affecting the Registration and License of Physicians of the State of New York

**Published:** 1890-08

**Authors:** 


### A NEW LAW AFFECTING THE REGISTRATION AND LICENSE OF PHYSICIANS OF THE STATE OF NEW YORK.

Chapter 500, of the laws of New York, 1890, materially modifies the preëxisting laws of registration and license, but affects only those physicians having been graduated or licensed by institutions or bodies outside this State, and who had not been authorized to practise in this State previous to the *twenty-fourth* day of June, 1890.

The full amendment is as follows :

All who, having been graduated from incorporated medical schools or colleges without the State as doctors of medicine, or licensed to practise physics or surgery under the laws of those European countries in which said degree does not confer the right so to practise, shall procure their diplomas from said corporations, or their licenses from such countries, to be indorsed by the regents of the university on the recommendation of a legally constituted board of medical examiners of this State. Every such indorsement shall be in form of Schedule A or of Schedule B, provided by the tenth section of this act. The regents shall keep a record of such indorsements, and may require applicants to verify their statements under oath; any indorsement made with fraudulent intent, or gross carelessness or ignorance, shall be deemed a misdemeanor, and shall subject the indorser or indorsers, upon conviction thereof, to a fine of two hundred and fifty dollars; provided, however, that no such indorsement as is above specified shall be made until the applicant thereof shall file with the person, officer or body above named as authorized to make such indorsement, or certificate, signed by the secretary of the University of the State of New York, showing that such applicant has received the degrees of bachelor or master of arts, of bachelor or master of science, or of bachelor or doctor of philosophy, from a college or university duly authorized to confer the same ; or that he has passed an examination conducted under the authority and in accordance with the rules of the Regents of the University of the State of New York, in arithmetic, grammar, geography, spelling, American history. English composition and elementary physics; or that he possesses qualifications which the regents have considered and accepted as fully equivalent to the above-named qualifications; as such degrees and certificate are more particularly defined in an act of the legislature of the State of New York, by chapter four hundred and sixty-eight of the laws of eighteen hundred and eighty-nine, entitled "An act to provide for the preliminary education of medical students," and as the same may be hereafter amended.

Thus, physicians not holding diplomas granted by some legally incorporated college of this State, and desiring to practise in this State must first possess the preliminary qualifications required of all medical students before graduation from a medical school of this State ; second, their diplomas must be indorsed by the Regents of the University of the State of New York, at Albany, upon the recommendation of a legally constituted board of medical examiners of this State; third, the then indorsed diplomas or licenses must, as heretofore, be registered in the County Clerk's office of the county in which they intend to practise in.

This new law seems, also, to take from the medical colleges of this State the power to endorse diplomas or licenses granted outside of the State.

Any person, therefore, who has begun the practice of medicine in this State, since June 24, 1890, and has not complied with the law as above, is undoubtedly practising illegally.

---

We reproduce the following editorial from the *Cincinnati Lancet-Clinic* of July 5, 1890, as a timely and considerate statement of questions that are of great professional interest and importance just at this time :

THE HEAT AND ITS EFFECTS.

The heat of the last two weeks has been remarkable as occurring so early in the season, it being very rare, indeed, for fatal sunstrokes to occur in the first summer month. More than a score have already taken place in this city, while the news of the daily press informs us of a similar mortality in other cities and towns, while even those who live in the country are not exempt from the fatal effects of the sun.

Reports of sunstroke are usually of the heat effects on adults, while the direct and indirect effects on the infant population are many times as great. Too often their main nutrient, milk, has become tainted or poisoned from the absorption of germs and gases, making of it a dangerous article of food, and productive of summer enteritis, or other trouble that leads to a fatal termination.

At this time of the year it is a good plan to have all milk sterilized as soon as possible. This is a very simple process, and consists of putting the milk in a clean bottle, loosely corking with a clean, new cork, and then placing the bottle in a vessel of water, and heating it slowly to the boiling point, this temperature being continued for forty-five minutes; then tightly cork the bottle and set it in a cool place until needed for use.

The nutrient properties of the milk are not destroyed, or even weakened, by this process, but for most persons it is more easily digested and is more nourishing.

Babies, children and adults, in hot weather, should live as much as possible in the shade, where there is the freest possible circulation of pure air. Long and frequent cool baths for infants are very conducive to their health and comfort. There is nothing like a long, cool bath, to relieve the discomfort of prickly or summer heat, following this with a little anointing of the creases of the skin with cold cream, vaseline, or fresh lard.

In cases of looseness of the bowels, a few doses of the ordinary chalk mixture will usually furnish the desired relief. This should be given in tablespoonful doses, and after every stool. Where there is a weakening of vitality, with very great propriety and advantage, teaspoonful doses of maltine may be added to the sterilized milk; the diastatic power of maltine being capable of rendering soluble and digestible any starchy food that may be in the stomach. Starch foods, such as Irish potatoes and breads, have often been regarded as the immediate and irritating cause of infantile enteric disorders. In part this may be true, and yet these starch foods were the very ones the lacteals and absorbents were crying for, and needed to stay the waste that was going on with fatal rapidity.

Right here the inestimable value of maltine, with its diastatic solvent properties, is quickly made manifest in changing the character of the discharges, and causing an irritant factor to become one of nutrition; given in sterilized milk the benefit of both is obtained.

In the city it is a good thing, in every possible case, to send the mother and infant out to the parks and suburbs for one, two, or three hours after sundown. The car ride is easy, while a shawl or other garment spread on the grass will afford a genuine relief and change from the mother's lap or cradle.

A little instruction from the family physician to his patrons in these simples may be the means of saving many valuable lives; nor should the physician take it for granted that his clients are informed in such matters, for very intelligent people sometimes are very ignorant of the plainest hygienic rules. This is especially the case in regard to the care of very young children. We recently saw an illustration of this in a very intelligent appearing mother, who did not even know how to hold her infant in positions of comfort to the babe and ease to herself. Even in such matters as this the doctor may give wholesome advice.

